# Iliopsoas Abscess Heralding the Diagnosis of Crohn's Disease in a Young Male

**DOI:** 10.1155/carm/5555016

**Published:** 2025-02-08

**Authors:** Majed Ali, Karam Karam, Emanuel-Youssef Dib, Lamia Azizi, Elias Fiani

**Affiliations:** ^1^Department of Internal Medicine, University of Balamand, Beirut, Lebanon; ^2^Department of Gastroenterology, University of Balamand, Beirut, Lebanon; ^3^Department of Radiology, University of Balamand, Beirut, Lebanon

**Keywords:** Crohn's disease, fistulous tract, iliopsoas abscess

## Abstract

Iliopsoas abscess (IPA) is a rare but potentially life-threatening complication that may occur in patients with Crohn's disease. We present the case of a 28-year-old male with Crohn's disease who developed a complicated IPA. Diagnosis was confirmed via CT imaging and colonoscopy, revealing a fistulous connection to the terminal ileum. The treatment involved percutaneous drainage (PCD), antibiotics, and infliximab. Timely diagnosis, appropriate imaging, and multidisciplinary care are critical to prevent morbidity and recurrence in patients with Crohn's disease complicated by IPA. This case highlights the importance of personalized treatment strategies and close follow-up in managing Crohn's-related IPA.

## 1. Introduction

Iliopsoas abscess (IPA) is an uncommon but potentially life-threatening condition, with an incidence that ranges from 0.4 to 4.2 cases per 100,000 individuals per year. It is characterized by pus accumulation within the iliopsoas muscle, a muscle located near vital structures such as the kidneys, ureters, and gastrointestinal tract, which predisposes it to infection from adjacent organs. Due to its vague clinical symptoms and insidious onset, diagnosis is often delayed, leading to high rates of morbidity and mortality [1]. The condition can be classified into two forms: primary, resulting from hematogenous spread, and secondary, which arises from a direct spread of infection from a nearby source [2].

Secondary IPA, accounting for most cases in developed countries, is often associated with gastrointestinal diseases, particularly Crohn's disease [3]. Crohn's disease is recognized as the most common cause of secondary IPA, given its ability to cause fistulae and abscesses in the surrounding tissues, which can easily spread to the iliopsoas muscle [4]. In a study by Hsieh et al. [3], it was shown that 76% of IPA cases were of secondary origin, with Crohn's disease being a leading contributor.

The clinical presentation of IPA can be highly variable, with symptoms such as fever, back pain, and limited hip movement forming the classical triad. However, this triad is seen in only about 30% of patients, making diagnosis difficult [4]. Imaging modalities, particularly computed tomography (CT), play a pivotal role in diagnosing IPA, with CT being the gold standard for its accuracy and ability to detect abscess characteristics such as gas formation and multiloculation [1]. Magnetic resonance imaging (MRI) is also useful, especially in cases where adjacent structures are involved [3].

Treatment typically involves a combination of antibiotics and drainage. Percutaneous drainage (PCD) is considered the treatment of choice due to its minimally invasive nature, but surgical intervention may be necessary in cases where PCD fails or when the abscess is gas-forming [1, 3]. In cases involving Crohn's disease, surgical management is often preferred, as it allows for simultaneous resection of the diseased bowel tissue and drainage of the abscess [4].

## 2. Case Presentation

**Figure 1:**
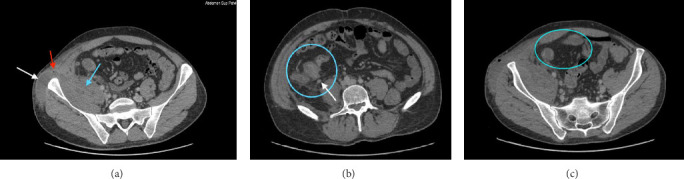
(a) Contrast-enhanced CT of the abdomen and pelvis showing signs suspicious of iliopsoas abscess secondary to Crohn's disease: white arrowhead–abscess extension. Red arrowhead—fistulous tract connecting the external collection of pus with the abscess at the terminal ileum (blue arrowhead). Only intravenous contrast medium had been used, and no oral contrast was given. (b) Contrast-enhanced CT of the abdomen and pelvis showing signs suspicious of iliopsoas abscess secondary to Crohn's disease: blue circle—thickening of terminal ileum. White arrowhead—enlarged lymph node. Only intravenous contrast medium had been used, and no oral contrast was given. (c) Contrast-enhanced CT of the abdomen and pelvis showing signs suspicious of iliopsoas abscess secondary to Crohn's disease: blue circle—thickening of terminal ileum. Only intravenous contrast medium had been used, and no oral contrast was given.

**Figure 2:**
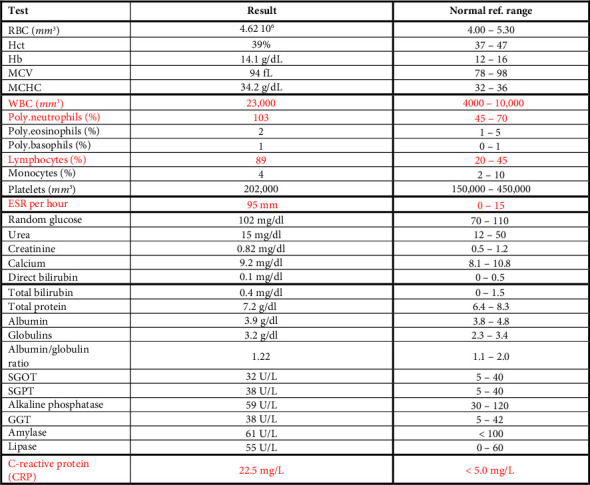
Patients' labs upon presentation to the ER.

A 28-year-old male patient presented to the emergency department with a persistent fever (39°C) unresponsive to self-administered acetaminophen. A review of systems (ROS) was negative, and there was no family history of Crohn's disease or inflammatory bowel disease (IBD). A CT scan of the abdomen and pelvis with low dose IV contrast was performed, revealing findings suggestive of Crohn's disease: “Significant submucosal thickening and enhancement at the level of the ileocecal valve and terminal ileum with surrounding fat stranding and multiple associated surrounding lymph nodes. A collection in the right iliopsoas/iliacus muscle extending through a fistulous tract to the subcutaneous soft tissues of the right lateral abdominal wall, where there is also a well-defined collection showing rim enhancement and few foci of air compatible with an abscess. There is the presence of a fistulous tract between the terminal ileum and iliopsoas about 1 cm from the ileocecal valve. The above described findings may represent a complicated Crohn's disease with fistula and abscess formation.” (Figure 1). Laboratory investigations (Figure 2) showed an increase in inflammatory markers and increased white cell count. Further tests showed a significantly elevated fecal calprotectin level of 900 μg/g.

Following these findings, the patient was scheduled for a colonoscopy. The colonoscopy revealed multiple superficial ulcerations at the terminal ileum, interspersed with areas of normal mucosa, with a characteristic cobblestone appearance. The disease was localized to the ileocecal valve, and biopsies confirmed the diagnosis of Crohn's disease, showing noncaseating granulomas and signs of cryptitis.

Subsequently, the patient underwent PCD of an associated abscess, which was sent for culture. Once the drainage had ceased, the percutaneous catheter was removed, and follow-up imaging demonstrated satisfactory resolution of the abscess. The patient was treated with infliximab at 5 mg/kg and antibiotics covering *Staphylococcus aureus* and enteric pathogens (vancomycin 20 mg/kg every 12 h and meropenem 1 g every 8 h) for 3 weeks.

At the 1-month follow-up, repeat imaging showed a significant reduction in the size of the abscess. The patient demonstrated clinical improvement and continued to respond well to the treatment plan.

## 3. Discussion

This case highlights the complexity of diagnosing and managing IPAs, particularly in the context of Crohn's disease. IPAs, though uncommon, can be life-threatening due to their nonspecific presentation, delayed diagnosis, and potential for severe complications such as sepsis or fistula formation. The association between Crohn's disease and secondary IPA is well-documented, as the transmural inflammation typical of Crohn's disease increases the likelihood of fistula formation, facilitating the spread of infection into the iliopsoas muscle [5, 6].

The clinical presentation of IPA in this patient was consistent with the literature, as symptoms such as fever and abdominal pain are common. However, the absence of a family history of Crohn's disease and the initially negative ROS could have delayed the diagnosis. This is not unusual, as the classic triad of IPA—fever, flank pain, and restricted hip movement—only occurs in about 30% of cases [6]. Moreover, the patient's fever and abdominal pain may have led clinicians to focus on more common intra-abdominal pathologies. As demonstrated by Guerrero et al. [5] and Abraham et al. [7], the nonspecific nature of IPA symptoms often leads to misdiagnosis or delayed recognition, which can worsen patient outcomes.

CT imaging played a pivotal role in this case by providing detailed insights into the abscess, fistula formation, and associated Crohn's disease features. CT remains the gold standard for diagnosing IPA due to its ability to delineate abscess characteristics, such as gas formation and multiloculation, both of which were present in this case [6, 8]. MRI, though useful in certain cases, was not employed here due to the need for a rapid, clear diagnosis to guide intervention. As Rogers et al. [8] noted in a similar case, CT imaging is often the most practical choice in emergent situations due to its availability and precision. One limitation of this case report is that an enteral contrast was not used during imaging, which would have further characterized the abscess and the fistulous tract.

Management of the abscess in this case involved PCD, which is typically the treatment of choice for its minimally invasive nature. However, given the involvement of Crohn's disease and the fistulous tract, surgical intervention may have been necessary if the abscess had not resolved satisfactorily with PCD alone [5, 8]. The addition of infliximab to manage the underlying Crohn's disease was crucial, as medical management of Crohn's not only prevents further complications but also reduces the likelihood of recurrent abscess formation [7, 8]. The success of infliximab and antibiotics in resolving the abscess and improving the patient's condition is consistent with the established literature, where biologic therapy has shown to induce remission in Crohn's patients with complicated disease [6].

Recurrent abscess formation is a known complication in Crohn's-related IPAs, and this case highlights the importance of close follow-up. As Rogers et al. demonstrated, patients with fistula-related abscesses are at increased risk for recurrence, necessitating both medical management and ongoing surveillance. Fortunately, in this case, the patient responded well to treatment, and follow-up imaging revealed significant improvement. Nonetheless, vigilance is warranted, as recurrence rates for Crohn's-related abscesses are high, and surgical resection of affected bowel segments may be required in future flare-ups [5, 8].

What makes this case unique is that the patient did not present any gastrointestinal symptoms that are peculiar to Crohn's disease; rather, his symptoms were related to his psoas abscess. This case report conveys a clear key clinical message that not every patient with Crohn's disease presents with diarrhea and or bloody stools. This manuscript serves as a reminder to keep Crohn's disease in the differential diagnosis when approaching a patient with an incidental finding of a psoas abscess on imaging.

In summary, this case underscores the challenges of diagnosing and managing IPAs in patients with Crohn's disease. The nonspecific presentation, combined with the insidious nature of the disease, necessitates a high degree of clinical suspicion, especially in young patients with vague abdominal and musculoskeletal symptoms. Prompt imaging, appropriate drainage, and biologic therapy targeting Crohn's disease are essential for achieving successful outcomes. Long-term monitoring and multidisciplinary management are also critical to prevent recurrence and manage Crohn's disease effectively.

## 4. Conclusion

This case highlights the complexity and potential severity of IPAs, particularly when secondary to Crohn's disease. It emphasizes the importance of early recognition and diagnosis, as delayed treatment can lead to significant morbidity. Imaging, particularly CT, remains a critical tool for identifying abscesses and fistula formation, while a combination of PCD, antibiotics, and biologic therapy, such as infliximab, proves to be an effective management strategy. As Crohn's disease significantly increases the risk of IPA recurrence, long-term monitoring and a multidisciplinary approach are essential for preventing complications and ensuring optimal patient outcomes. This case also underscores the necessity of individualized treatment plans that address both the abscess and the underlying Crohn's disease to prevent recurrence and further complications.

## Data Availability

Data will be made available upon request from authors.
